# Clade Ib Mpox Outbreak — Kenya, July 2024–February 2025

**DOI:** 10.15585/mmwr.mm7422a2

**Published:** 2025-06-19

**Authors:** Pius Mutuku, Ahmed Abade, Maurice Owiny, Zephania Irura, Abdi Roba, Hilary Limo, Amy Herman-Roloff, Naomi Lucchi, Nancy Bowen, Ahmed Fidhow, Daniel Lang’at, Jonas Z. Hines

**Affiliations:** ^1^Kenya Field Epidemiology and Laboratory Training Program, Nairobi, Kenya; ^2^Africa Field Epidemiology Network, Nairobi, Kenya; ^3^Division of Disease Surveillance and Response, Ministry of Health, Nairobi, Kenya; ^4^National Public Health Laboratory, Ministry of Health, Nairobi, Kenya; ^5^Public Health Emergency Operations Centre, Ministry of Health, Nairobi, Kenya; ^6^Division of Global Health Protection, CDC Kenya, Nairobi, Kenya.

**Summary**

**What is already known about this topic?**

Since July 2024, Kenya has been experiencing an mpox outbreak caused by clade Ib *Monkeypox virus*, a newly recognized subclade.

**What is added by this report?**

Among 48 laboratory-confirmed clade Ib mpox cases diagnosed in Kenya during July 2024–February 2025, a total of 27 (56.3%) occurred among persons who worked as truck drivers, or were in contact with them, along a highway from Mombasa to Malaba, a transportation corridor that links Kenya to other East and Central African countries. Two thirds (30; 63%) of the cases were likely to have been sexually transmitted. Eleven (23%) patients also had HIV infection, one of whom died.

**What are the implications for public health practice?**

Public health measures, including vaccination focusing on those most at risk for mpox such as truck drivers, sex workers, and persons traveling to countries with ongoing clade Ib mpox outbreaks, might help stop the spread of the disease within Kenya and to other countries.

## Abstract

Since July 2024, Kenya has been experiencing an mpox outbreak caused by clade Ib *Monkeypox virus (*MPXV), a newly recognized variant that has spread from the Democratic Republic of the Congo to multiple countries within and outside of Africa. This report describes the characteristics of laboratory-confirmed clade Ib mpox cases in Kenya during the first 7 months of the outbreak. Among 447 suspected cases during July 2024–February 2025, a total of 48 (10.7%) were confirmed by polymerase chain reaction testing. Most confirmed cases occurred along a highway from the Indian Ocean port in Mombasa to Malaba at the Ugandan border, a transportation corridor that links Kenya to other East and Central African countries. Among the 48 confirmed cases, 27 (56.3%) occurred among persons associated with the transportation corridor, including truck drivers (12; 25.0%), sex workers (eight; 16.7%), and persons employed at or near trucking stopovers (seven; 14.6%). Sexual transmission was suspected in 30 (62.5%) cases, based on the patient’s history or locations of the lesions; 11 (22.9%) patients also had HIV infection, one of whom died. Clade Ib MPXV in Kenya appears to be primarily sexually transmitted and concentrated in specific groups at high risk for infection. Public health measures, including vaccination, might be most effective if they focus on these specific groups and geographic areas.

## Introduction

Mpox is an emerging global public health threat, with outbreaks primarily caused by clade II *Monkeypox virus* (MPXV) reported from multiple countries since 2022 (*1*–*3*). Since late 2023, the eastern region of the Democratic Republic of the Congo (DRC) has reported an increase in cases caused by a newly recognized subclade, clade Ib MPXV. This strain has spread primarily through sexual transmission in countries that have not previously reported mpox cases (*3*,*4*). In August 2024, the Africa Centres for Disease Control and Prevention and the World Health Organization both escalated the clade Ib mpox outbreak to their highest respective public health threat levels.* Although clade I MPXV has historically been associated with small outbreaks in Central Africa after zoonotic transmission in rural forested areas with limited human-to-human spread, sustained human-to-human transmission appears to be an important characteristic of this outbreak (*5*,*6*). Information on the epidemiologic characteristics of clade Ib mpox is limited. Kenya confirmed its first clade Ib mpox case on July 29, 2024.^†^ This report describes the characteristics of patients with confirmed mpox cases during the first 7 months of the clade Ib outbreak. These findings can be used to guide response strategies.

## Methods

### Data Sources

Epidemiologic and clinical information was extracted from mpox outbreak investigation documents for July 29, 2024, through February 28, 2025, including case investigation forms, outbreak investigation reports, and information from health care facilities about characteristics of patients with confirmed cases. Persons with suspected infection identified at health care facilities or through contact tracing investigations were isolated, either at a health facility or at their homes, and interviewed by public health workers using a standardized questionnaire to collect demographic, clinical, and behavioral information. Contact tracing was conducted for persons with confirmed mpox cases to identify potential secondary cases. A suspected mpox case was defined as the occurrence of an unexplained rash and at least one constitutional symptom (headache, fever of >101.3°F [>38.5°C], lymphadenopathy, myalgia, back pain, or asthenia) in a person with either recent (within the preceding 3 weeks) travel to a country experiencing an mpox outbreak or contact with such a person within the preceding 3 weeks. A confirmed case was defined as a suspected case with a positive MPXV polymerase chain reaction (PCR) or genomic sequencing result. MPXV PCR testing was performed at the Kenya National Public Health Laboratory.^§^ The MPXV clade was confirmed through genomic sequencing (*7*) at the Kenya Medical Research Institute/Walter Reed Army Institute of Research laboratory in Kisumu.

### Exposure Categories

MPXV exposures were categorized as suspected sexual contact, nonsexual contact, or unknown contact. Cases suspected to be sexually transmitted were those diagnosed in a person who reported a new sexual partner or who engaged in transactional sex during their incubation period and those for which the investigation suggested exposure through sex (e.g., a symptomatic sexual partner or primary lesions in the genital area). Nonsexually transmitted cases were defined as those for which the investigation did not suggest sexual transmission (e.g., a case in a child living in the same household). The exposure category for cases that were not classified as sexually or nonsexually transmitted were classified as unknown. A prodrome was defined as experiencing any signs or symptoms (e.g., fever or headache) at least 1 day before rash onset.

### Analysis

Descriptive analyses of confirmed cases^¶^ were performed in R (version 4.4.2; R Foundation). The Kenya Ministry of Health (MOH) and county health departments granted permission to conduct investigations as part of an ongoing public health emergency, and patients consented to interviews and MPXV testing. This activity was reviewed by CDC, deemed not research, and conducted consistent with applicable federal law and CDC policy.**

## Results

### Characteristics of Patients with Confirmed Clade Ib Mpox

**FIGURE 1:**
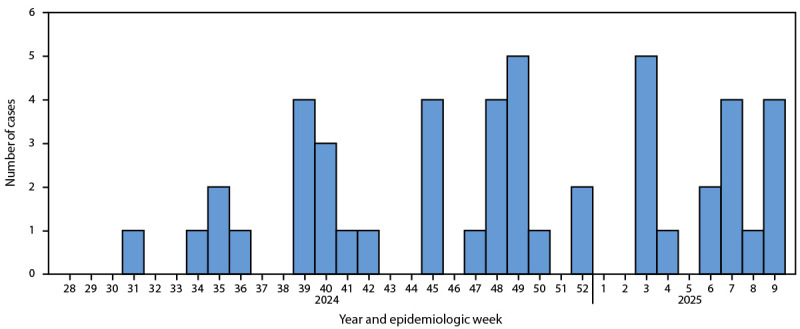
Number of clade Ib mpox cases, by week of confirmation (N = 48) — Kenya, July 2024–February 2025

**FIGURE 2:**
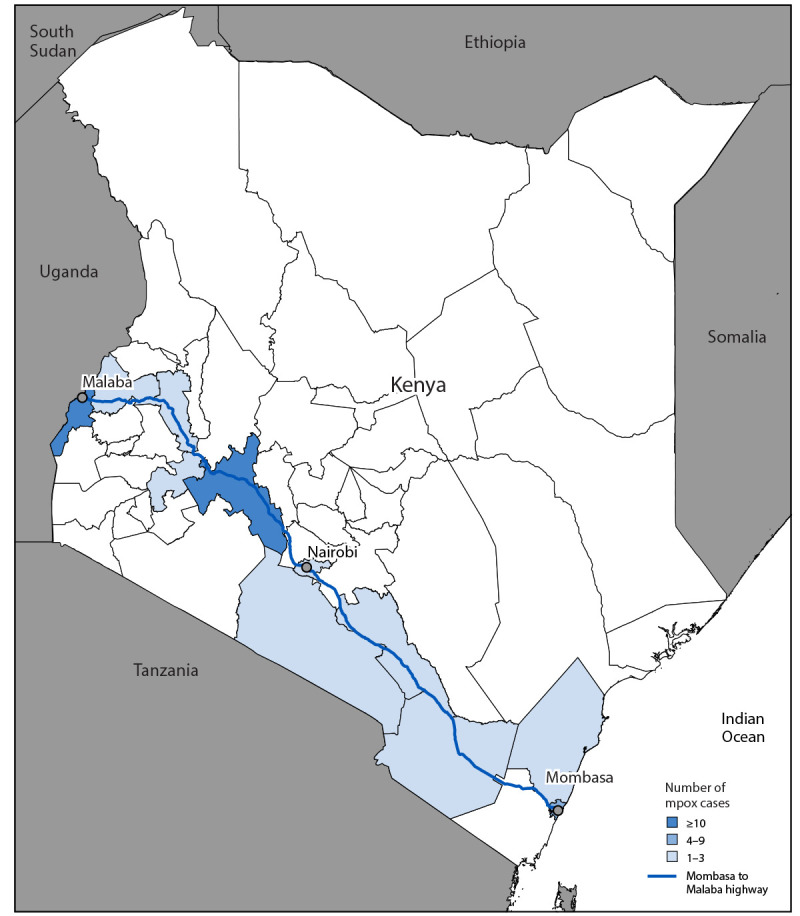
Number of confirmed clade Ib mpox cases, by county of report and proximity to the highway from Mombasa to Malaba — Kenya, July 2024–February 2025* * Number and percentage of cases, by county: Bungoma: three (6%); Busia: 12 (25.0%); Kajiado: two (4%); Kericho: two (4%); Kilifi: two (4%); Makueni: two (4%); Mombasa: nine (19%); Nairobi: three (6%); Nakuru: 10 (21%); Taita Taveta: two (4%); Uasin Gishu: one (2.1%).

Among 447 suspected cases tested by PCR during July 2024–February 2025, a total of 48 (10.7%) were confirmed positive. All confirmed clade Ib mpox cases in Kenya (Figure 1) were detected along a transportation corridor that connects Kenya’s international southeastern seaport in Mombasa with Malaba, on the country’s western border with Uganda (Figure 2). Among the 48 persons with confirmed mpox, 28 (58.3%) were women, and the median age was 35.0 years (IQR: 29.0–38.0 years) (Table). Three (6.3%) were children aged ≤14 years. Eleven (22.9%) patients had HIV infection, all of whom had received some HIV treatment. A prodrome was reported by 17 (35.4%) patients. All persons with confirmed cases developed a generalized body rash, and 33 (68.7%) had genital lesions. Most patients had at least one health care visit for their illness before mpox was suspected, including 24 (50.0%) with one visit and 16 (33.3%) with two or more visits. The median interval between rash onset and laboratory confirmation was 7.5 days (IQR: 5–13 days).

**TABLE:** Demographic and clinical characteristics of persons with confirmed clade Ib mpox (N = 48) — Kenya, July 2024–February 2025

Characteristic	No. (%)
**Sex**	
Female	28 (58.3)
Male	20 (41.6)
**Age group, yrs**	
0–14	3 (6.3)
15–29	6 (12.5)
30–44	36 (75.0)
≥45	3 (6.3)
**Occupation**	
Truck driver	12 (25.0)
Sex worker	8 (16.7)
Employee at or near a truck stopover	7 (14.6)
Other occupation	9 (18.8)
Housewife	6 (12.5)
None (e.g., child)	6 (12.5)
**HIV status**	
Negative	11 (22.9)
Positive	11 (22.9)
Unknown	26 (54.2)
**History of international travel**	
Yes*	18 (37.5)
No	27 (56.2)
Unknown	3 (6.3)
**Suspected sexual transmission**	
Yes	30 (62.5)
No	5 (10.4)
Unknown	13 (27.1)
**No. of health care visits before mpox suspected**	
0	3 (6.3)
1	24 (50.0)
≥2	16 (33.3)
Unknown	5 (10.4)
**No. of days from symptom onset^†^ to laboratory confirmation**	
≤2	3 (6.3)
3–5	15 (31.3)
6–9	11 (22.9)
≥10	19 (39.6)
**No. of close contacts^§^**	
0	0 (—)
1–4	9 (18.8)
5–9	9 (18.8)
10–19	8 (16.7)
≥20	8 (16.7)
Unknown	14 (29.2)
**Prodrome present**	
Yes	17 (35.4)
No	31 (64.6)
**Signs and symptoms**	
Generalized rash	48 (100)
Fever	41 (85.4)
Genital lesions	33 (68.7)
Headache	20 (41.7)
Sore throat	19 (39.6)
Cough	14 (29.2)
Lymphadenopathy	7 (14.6)
**Outcome**	
Recovered (one fetal death)^¶^	47 (97.9)
Death**	1 (2.1)

* Countries visited included the Democratic Republic of the Congo, Rwanda, Tanzania, and Uganda, all of which are experiencing clade Ib mpox outbreaks at the time of this report.

^†^ If a prodrome was present (any signs or symptoms such as fever or headache at least 1 day before rash onset), the date of rash onset was used.

^§^ Number of close contacts was not collected for six cases.

^¶^ A pregnant woman with confirmed mpox recovered but delivered a stillborn fetus at 38 gestational weeks.

** One patient died 30 days after rash onset. The patient, who had advanced HIV disease, was hospitalized for cryptococcal meningitis and had secondarily superinfected anogenital lesions and severe constipation.

### Outcomes of Patients with Confirmed Clade Ib Mpox 

One patient died. The patient had advanced HIV disease and was not receiving antiretroviral therapy.^††^ The death occurred 30 days after rash onset while the patient was hospitalized for cryptococcal meningitis and had secondarily superinfected anogenital lesions and severe constipation. In addition, a pregnant woman delivered a stillborn fetus at 38 gestational weeks. All other patients recovered.

### Potential Sources of Exposure 

Among patients with confirmed cases, 18 (37.5%) reported travel to nearby countries experiencing active clade Ib mpox outbreaks during their incubation period (i.e., DRC, Rwanda, Tanzania, or Uganda); five (10.4%) did not have an epidemiologic link to any other cases or report recent travel. Twelve cases (25.0%) occurred in truck drivers; eight (16.7%) in sex workers, all of whom reported having clients who were truck drivers; and seven (14.6%) in persons employed at or near truck stopovers. Genomic sequencing of specimens from 33 patients with confirmed mpox identified the circulating strain as clade Ib MPXV (Kenya MOH, Mpox Situation Report 180, unpublished data, March 3, 2025).

MPXV was suspected to have been transmitted through sexual contact for 30 (62.5%) patients; other means of transmission included close, nonsexual household contact (five; 10.4%) and unknown means (13; 27.1%). The median number of close contacts per case was 7.5 persons (IQR: 4.3–15.8). Among 490 identified close contacts, 10 secondary cases were identified (secondary transmission rate: 2.0%).

### Public Health Response

The Kenya MOH led the mpox outbreak response. MOH activated the Kenya Public Health Emergency Operations Center (EOC), an incident management system, after detecting the index case, and affected counties activated their county-level emergency operations centers. Field epidemiologists investigated confirmed cases and conducted contact tracing. Persons with a diagnosis of confirmed mpox were isolated in hospitals or their homes. MOH heightened its mpox surveillance, including developing and circulating case definitions and clinical management guidelines for frontline health workers across Kenya. MOH also intensified screening measures at points of entry^§§^ for travelers with mpox signs and symptoms, linking travelers with suspected cases to public health workers, as well as increased distribution of mpox information. Laboratory systems were strengthened with MPXV PCR capability established in four laboratories in Kenya, and genomic sequencing was used to confirm the clade (*7*). Kenya instituted public awareness efforts by briefing the media, distributing educational materials, and meeting with affected groups and the public. Kenya requested and received mpox vaccine as part of the Access and Allocation Mechanism and is planning a July 2025 vaccination campaign for the highest risk groups.^¶¶^ Through the EOC, MOH collaborated with other Kenyan government agencies and multilateral entities to respond to the mpox outbreak.

## Discussion

A clade Ib mpox outbreak has been occurring in Kenya since July 2024. Most confirmed cases occurred in persons associated with the transportation corridor that links Kenya to other East and Central African countries, many of which have ongoing clade Ib mpox outbreaks. The interaction between truck drivers and sex workers might play a role in mpox transmission in this country. Evidence of secondary transmission appears limited; however, the risk for ongoing transmission remains because of the country’s regional commercial connectivity. Widespread mpox in Kenya could directly affect the health care system, which is already managing an HIV epidemic; HIV is a risk factor for severe mpox (*8*). Although HIV affects the general population in Kenya, certain populations are disproportionately affected, including sex workers. The one reported death associated with clade Ib mpox in Kenya occurred in a person with HIV infection that was complicated by an opportunistic infection. Public health interventions, including mpox vaccination, that focus on groups at high risk for infection could be important to mitigating additional spread and limiting morbidity and mortality.

Kenya’s mpox outbreak highlights the challenges of controlling infectious disease outbreaks in highly connected geographic regions. Multiple mpox cases in persons who traveled to countries with widespread clade Ib transmission and who had no epidemiologic links to other cases in Kenya suggest that multiple introductions are occurring in the country. However, the identification of cases in persons with no travel history or epidemiologic link to other cases also suggests undetected community transmission in Kenya. Genomic epidemiology might supplement epidemiologic findings from case investigations by providing insights into potential transmission networks among cases that otherwise seem unrelated. Whereas many initial infections appear to have been acquired through sexual contact, subsequent transmission within households can occur if appropriate isolation measures are not implemented. 

During the first 7 months of the clade Ib mpox outbreak in Kenya, the country documented approximately 500 MPXV tests. Although MOH has circulated case definitions and trained health care workers in mpox diagnosis, case management, and infection prevention, the testing numbers are low, possibly reflecting lack of knowledge about mpox in the general public and among health care workers. Some patients made several health care visits before mpox was suspected. The differential diagnosis of mpox is broad. One of the primary conditions that confounds diagnosis is infection with varicella (chickenpox) (*9*,*10*), which causes seasonal outbreaks in Kenya. Additional outreach to increase awareness of mpox signs and symptoms could facilitate timely detection of cases and isolation of patients. Although multiple laboratories in Kenya can conduct molecular testing, mpox testing was only available in four laboratories in Nairobi and Kisumu during the period in this report. Expanding mpox laboratory diagnostics in other geographic areas and using rapid diagnostic tests with adequate sensitivity could also reduce the time between symptom onset and mpox confirmation. Vaccinating persons highest at risk for acquiring mpox might help halt transmission within Kenya and to other countries.

The increasing numbers of mpox cases globally in recent years is likely a result of many factors. These include increases in mpox exposures (i.e., increases in sexual transmission, ease of international travel, and encounters with animal reservoirs), improvements in disease surveillance, and decreasing population-level immunity to orthopoxviruses, the genus that includes MPXV and *Variola virus* (smallpox), as the cohort vaccinated against smallpox ages (*3*).

### Limitations

The findings in this report are subject to at least two limitations. During outbreaks, staff members responsible for data collection often are also responding to other demands of the public health emergency, which can affect the completeness and quality of the data. Second, surveillance and laboratory gaps might have resulted in missed cases and an overall underestimation of total number of cases.

### Implications for Public Heath Practice

As the mpox outbreak evolves in Kenya, public health measures focusing on interventions to interrupt transmission are critical, such as vaccination of persons at highest risk for infection, including truck drivers and sex workers (preexposure prophylaxis) and close contacts of mpox patients (postexposure prophylaxis).*** The strengths of Kenya’s existing HIV program could be used to improve risk communication and outreach efforts and minimize mpox-associated morbidity and mortality among groups disproportionately affected by both viruses. Public health messaging focusing on sexual and nonsexual transmission also are important for controlling the outbreak. These interventions could help protect populations at risk for severe mpox, including those living with HIV, and help prevent mpox transmission to other countries.

## Footnotes

* Africa CDC | Africa Declares Mpox a Public Health Emergency of Continental Security, Mobilizing Resources Across the Continent; World Health Organization | WHO Director-General Declares Mpox Outbreak a Public Health Emergency of International Concern

† Other countries with sustained transmission of clade Ib mpox: Burundi, Democratic Republic of the Congo, Kenya, Malawi, Rwanda, South Sudan, Tanzania, Uganda, and Zambia (CDC | Mpox in the United States and Around the World: Current Situation). Sustained transmission was defined as at least 10 confirmed cases associated with community transmission reported during the past 6 weeks, or alternatively, one to nine confirmed cases associated with community transmission reported from at least four geographic locations.

§ Specimens were collected by swabbing lesions on least two body locations of each patient. Each patient’s swabs were placed together in the same media and transported to the National Public Health Laboratory in Nairobi.

¶ Four children who were household contacts of a single person with a confirmed case received positive MPXV test results (specimens taken via nasopharyngeal swab) but remained asymptomatic. PCR cycle threshold values were high (indicating low virus levels); no serologic testing was conducted to assess for evidence of MPXV exposure. The four children were not included in the analysis because their test results were suspected to be a result of environmental or laboratory contamination, which has previously been documented with MPXV (CDC | CDC’s Laboratory Outreach Communication System (LOCS). 08/23/2022: Lab Advisory: Mpox virus testing considerations to prevent false positive test results).

** 45 C.F.R. part 46, 21 C.F.R. part 56; 42 U.S.C. Sect. 241(d); 5 U.S.C. Sect. 552a; 44 U.S.C. Sect. 3501 et seq.

†† The patient who died met World Health Organization stage 4 classification criteria for a severe opportunistic infection that indicates significant immune suppression (World Health Organization | WHO case definitions of HIV for surveillance and revised clinical staging and immunological classification of HIV-related disease in adults and children). The official cause of death was not available at the time of this report.

§§
World Health Organization | Considerations for Border Health and Points of Entry for Mpox

¶¶
Republic of Kenya, Ministry of Health | Kenya Receives 10,700 Doses of Mpox Vaccines for the High Risk Population

***
World Health Organization | Mpox
